# Real-world data survival outcomes of first line immunotherapy with pembrolizumab in high expressers with stage IV non-small cell lung cancer

**DOI:** 10.2478/raon-2026-0050

**Published:** 2026-09-07

**Authors:** Vladimir Stojsic, Bojan Zaric, Petar Simurdic, Tomi Kovacevic, Darijo Bokan, Jelena Djekic Malbasa, Tatjana Sarcev, Goran Stojanovic, Daliborka Bursac, Borislava Nikolin

**Affiliations:** 1 Faculty of Medicine, University of Novi Sad, Novi Sad, Serbia; 2 Clinic for Thoracic Oncology, Institute for Pulmonary Diseases of Vojvodina, Sremska Kamenica, Serbia; 3 Faculty of Pharmacy, University Business Academy in Novi Sad, Novi Sad, Serbia; 4 Oncology Institute of Vojvodina, Sremska Kamenica, Serbia

**Keywords:** lung cancer, NSCLC, PD-L1, immunotherapy, pembrolizumab

## Abstract

**Background:**

Pembrolizumab monotherapy is standard of care in first line treatment of patients with stage IV lung cancer and programmed death ligand 1 (PD-L1) expression ≥ 50%. Whether the higher levels of PD-L1 expression predict higher benefit from the treatment with pembrolizumab is still debatable.

**Patients and methods:**

This trial analyzed clinical and pathological characteristics, overall response rate (ORR), and survival outcomes in patients with non-small cell lung cancer (NSCLC) and PD-L1 expression of ≥ 50% who received front line treatment with pembrolizumab in real-world setting. The aim was to evaluate does the level of expression influence survival endpoints.

**Results:**

Fifty-one consecutive patients were evaluated in this study. The median PFS for patients in this study was 12.0 months (95% CI, 7.7–16.2 months), and the median OS was 21.0 months (95% CI, 9.6–32.3 months). Based on PD-L1 status patients were divided into two groups. First one with PD-L1 expression of 50%–79%, and the second with expression over 80%. Gender distribution between the two groups (p = 0.029) and difference in stage of the disease (p = 0.039) were statistically significant. There was no significant difference in progression free survival (p = 0.548) and overall survival (p = 0.153) between two groups.

**Conclusions:**

This trial did not confirm relation between higher expression of PD-L1 and better survival outcomes. However, the pitfall of the trial was relatively small number of patients, so with the expansion of this real-world data (RWD) dataset future results might differ.

## Introduction

Lung cancer remains among deadliest cancers in both genders worldwide. It is the second most frequently diagnosed cancer and the leading cause of cancer-related deaths in 2022. One in ten newly diagnosed cancers worldwide is lung cancer, and one in five cancer related deaths are due to lung cancer.^1,2^ The incidence and mortality from lung cancer is twice as high in men compared to women. Male-to-female ratio, however, varies on geographic location. Five-year survival of patients with lung cancer still lingers from 10–20% globally.^2^ Serbia ranks second in the world considering morbidity and mortality of lung cancer.^3^ Based on the latest official data from Serbia (published in 2019), in terms of morbidity and mortality, lung cancer is first among males, while it holds second place for females, after breast cancer. According to this data, the largest number of affected individuals are aged between 65 and 69 years.

In more than 75% of patients, at the time of diagnosis, the disease is in advanced or metastatic stage. Moreover, the prognosis of lung cancer is strongly correlated with the stage of the disease, so the 5-year survival for metastatic stage (stage IV) is less than 5%.^4^

Advances in molecular biology and immunology of tumors, led to better understanding of underlying tumor growth and spread mechanisms, enabling new therapeutic options to improve survival outcomes. The turning point for development of active immunotherapies in lung cancer was understating of programmed death receptor 1 (PD-1) on T cells, and programmed death ligands 1 and 2 (PD-L1, and PD-L2) on tumors cells. That research created new class of drugs – immune check point inhibitors (ICIs).^5-9^

One of the pivotal drugs for lung cancer treatment, pembrolizumab, was initially tested in a large multicohort phase I study (KEYNOTE-001), followed by phase 2/3 study KEYNOTE-010.^10,11^ Both trials defined PD-L1 expression on tumors cells as an important predictor of response to therapy. Tumors with high PD-L1 expression (defined as ≥ 50% of tumor cells with membranous expression) were more likely to respond better to pembrolizumab.^9^

Although the cut off level for low PD-L1 expression (1%–49%) and high PD-L1 expression (50%–100%) is arbitrarily created, PD-L1 represents a continuous biomarker (with a range of 0% to 100%) and has been shown through studies to correlate with the effectiveness of immunotherapy.^10-13^ Theoretically, the higher the level of PD-L1 expression, the better the response to immunotherapy. There is still not enough clear and consistent data to confirm that patients with the highest level of PD-L1 expression would really have better survival outcomes on checkpoint inhibitor therapy. In clinical practice, the selection of patients with non-small cell lung cancer for immunotherapy is performed in relation to the level of PD-L1 expression. The expression of PD-L1 on the tumor cells, determined immunohistochemically (IHC), is currently the only diagnostic test approved by the Food and Drug Administration (FDA) and European Medicines Agency (EMA) for the selection of patients with non-small cell lung cancer (NSCLC) in relation to type of immunotherapy. Most investigators and physicians are aware of the imprecise connection between PD-L1 expression and survival outcomes in NSCLC, waging the benefit of treatment in relation to other, clinical and pathological prognostic factors.^14^

The effectiveness of pembrolizumab as monotherapy of metastatic NSCLC and a PD-L1 expression ≥ 50%, still needs to be additionally evaluated outside of the clinical trial setting, in real-world clinical practice - delivering real-world data for further analysis.

The primary aim of current study was to determine the duration of progression-free survival (PFS) and the duration of overall survival (OS) in patients with PD-L1 expression ≥ 50% treated with pembrolizumab immunotherapy in the first line of treatment. The trial also aimed to investigate whether there is a significant difference in clinical-pathological characteristics, progression-free survival time, and overall survival between patients with PD-L1 expression of 50%–79%and patients with PD-L1 expression of 80%–100%.

Major limitation of the trial is a smaller sample; however, it is a potential cornerstone for comparison between other smaller real-world data (RWD) datasets. While datasets expand further, evaluations might define the size of the datasets necessary for compiling real-world evidence.

## Patients and methods

This retrospective study included data from 51 consecutive patients who were diagnosed with metastatic NSCLC and who started treatment in Institute for pulmonary diseases of Vojvodina in period between 1^st^ January 2019 and 31^th^ December 2021. Patient data were collected from the institutional lung cancer registry and available electronic medical records. Patients eligible for participation in the study were those diagnosed with metastatic-stage non-small cell lung cancer without Epidermal Growth Factor Receptor (EGFR) gene mutations and Anaplastic Lymphoma Kinase (ALK) rearrangements, with PD-L1 expression ≥ 50%, and who had received at least one cycle of pembrolizumab monotherapy in the first line of treatment.

The study was conducted in accordance with the Declaration of Helsinki and approved by the Review and Ethics Committee of the Institute for Pulmonary Diseases of Vojvodina, Sremska Kamenica, Serbia (approval number: No 521/4 and 19-II/4 - 01.03.2023).

Clinical data were collected from the included participants, such as gender, age, smoking status, body mass index, Eastern Cooperative Oncology Group (ECOG) performance status, tumor histology, TNM disease stage, PD-L1 expression levels, duration of pembrolizumab treatment, and number of administered cycles. The radiological response to therapy was determined by experienced thoracic radiologists, by immune Response Evaluation Criteria In Solid Tumors (iRECIST) criteria version 1.1. TNM staging was done using the eighth edition TNM stage classification for lung cancer.

Progression-free survival (PFS) was defined as the time from the start of pembrolizumab treatment until the date of disease progression or death, whichever occurred first. Overall survival (OS) was defined as the time from the start of pembrolizumab treatment until death. Patients who were alive at the time of statistical analysis were censored at the date of their last contact.

PD-L1 expression was determined immunohistochemically (IHC) following institutional clinical practice and expressed as the percentage of tumor cells showing positive membrane staining. IHC assay used for analysis was Dako PD-L1 22C3 antibody. PD-L1 expression values were also collected from institutional electronic data capture system.

The data analysis included methods of descriptive and inferential statistics. Numerical attributes were presented using average values (arithmetic mean, median) and measures of variability (range, standard deviation), while categorical attributes were represented using frequencies and percentages. Univariate analysis involved *χ2* tests for categorical attributes and Student’s t-tests and oneway analysis of variance (ANOVA) for numerical attributes. Multivariate analysis included Cox regression and logistic regression analysis. The relationship between variables was determined using correlation analysis. The significance level of p < 0.05 was considered statistically significant. Eventtime variables (PFS and OS) were calculated using the Kaplan-Meier method. IBM SPSS Statistics 21 software package was used for statistical data processing.

## Results

This study included clinical data from patients with metastatic non-small cell lung cancer whose tumors have PD-L1 receptor expression of ≥ 50% and who received pembrolizumab as first-line treatment. Among the total of 51 patients, 31 (60.8%) were male, and 20 (39.2%) were female. The average age of the patients was 66.8 years (range 50–84 years). Most patients were smokers, accounting for 39 (76.5%). The median value of the packyear index was 38.2 (packs per year). Regarding Body Mass Index (BMI), the average value was 25.4. Most patients fell into the normal weight group, 22 (43.1%), and the overweight group, 23 (45.1%). Almost all patients in our study had an ECOG PS 1, 50 patients (98.0%). Clinical characteristics are given in Table 1.

**TABLE 1.** Clinical characteristics of patients with NSCLC

Characteristics	Frequencies N/(%)
**Gender**	
Male	31 (60.2%)
Female	20 (39.8%)
**Age (years)**	
Average	66.8
Range	50–84
**Smoking status**	
Non-smokers	3 (5.9%)
Active smokers	39 (76.5%)
Former smokers	9 (17.6%)
**Pack-year index**	
Average	48.5
Range	0–150
**Body mass index (BMI)**	
Average	25.4
< 18.5	3 (5.9%)
18.5–24.9	22 (43.1%)
25.0–29.9	23 (45.1%)
30.0–34.9	1 (2.0%)
> 40.0	2 (3.9%)
**ECOG performance status**	
0	1 (2.0%)
1	50 (98.0%)

1 ECOG = Eastern Cooperative Oncology Group; NSCLC = non-small cell lung cancer

Twenty-nine patients (56.9%) had tumors with non-squamous histology, all of which were classified as adenocarcinomas. Squamous cell carcinoma was confirmed in 22 patients (43.1%). In terms of TNM and disease staging, 36 patients (70.6%) were in stage IVA, while 15 patients (29.4%) were in stage IVB. The distribution of stages and histological classification is given in Table 2.

**TABLE 2.** Pathological characteristics and TNM staging

Histological subtype of NSCLC					
Squamous					22 (43.1%)
Non-squamous (adenocarcinoma)					29 (56.9%)
**TNM classification**					
T		N		M	
T1a	1 (2.0%)	0	10 (19.6%)	1a	20 (41.2%)
T1b	3 (5.9%)	1	3 (5.9%)	1b	16 (31.4%)
T1c	3 (5.9%)	2	20 (39.2%)	1c	15 (27.5%)
T2a	2 (3.9%)	3	18 (35.3%)		
T2b	6 (11.8%)				
T3	7 (13.7%)				
T4	29 (56.9%)				
**Stage**					
IVA		IVB			
36 (70.6%)		15 (29.4%)			

1 NSCLC = Non-Small Cell Lung Cancer; T = Tumor; N = Node; M = Metastasis

The largest number of patients had PD-L1 expression levels between 90%–100% The distribution of PD-L1 expression level is given in Table 3. Across the entire cohort, the median PD-L1 expression was 80% (range: 50–100%), and the average PD-L1 expression value was 76.5%.

**TABLE 3.** Levels of PD-L1 expression

PD-L1 expression N/(%)	
50–59%	5 (9.8%)
60–69%	11 (21.6%)
70–79%	9 (17.6%)
80–89%	13 (25.5%)
90–100%	13 (25.5%)

1 PD-L1 = Programmed Death-Ligand 1

All patients received the standard dose of 200 mg of pembrolizumab every three weeks until disease progression or the occurrence of unacceptable toxicity. The average duration of pembrolizumab treatment was 18.3 months (range: 2–60 months). The average number of treatment cycles with pembrolizumab was 21.3 (range: 2–61 cycles). (Table 4)

**TABLE 4.** Duration of treatment and number of cycles with pembrolizumab

DURATION OF TREATMENT (months)	
Average	18.3
Range	2–60
**NUMBER OF CYCLES WITH PEMBROLIZUMB**	
Average	21.3 cycles
Range	2–61 cycles

Control CT scans were performed every three months, with radiological responses evaluated according to the iRECIST 1.1 criteria. Among the patients included in this study, the majority showed stable disease (SD) as the best response to pembrolizumab therapy, accounting for 26 patients (51.0%). Radiological response, including ORR is given in Table 5.

**TABLE 5.** Radiological response to treatment according to the iRECIST criteria

(immuno) RADIOLOGICAL RESPONSE TO TRETMENT (iRECIST)	
Complete response (CR)	0 (0%)
Partial response (PR)	19 (37.3%)
Stable disease (SD)	26 (51.0%)
Progressive disease (PD)	6 (11.8%)
Overall response rate (ORR)	37.2%

1 iRECIST = (immuno) Response Evaluation Criteria In Solid Tumors

Progression-free survival (PFS) was defined as the time from the start of pembrolizumab treatment to the date of disease progression or death, whichever occurred first. Overall survival (OS) was defined as the time from the initiation of pembrolizumab treatment to the time of death. Patients who were still alive at the time of the analysis were censored at the date of their last contact. The median progression-free survival (mPFS) for patients in this study was 12.0 months (95% CI, 7.7–16.2 months), and the median overall survival (mOS) was 21.0 months (95% CI, 9.6–32.3 months). Kaplan Meyer survival curves for PFS and OS are given in Tables 1 and 2 respectively.

Given the distribution of PD-L1 expression in this cohort, patients were divided into two groups. The median PD-L1 expression value of 80% was used as the threshold for dividing the groups, ensuring that the number of patients in each group was as balanced as possible. Thus, one group consisted of patients with tumor PD-L1 expression of 50%–80%, while the other included patients with tumor PD-L1 expression of 80%–100%. The average age of patients with PD-L1 expression values of 50%–80% was 65.4 years, while for those with PD-L1 expression values of 80%–100%, it was 68.2 years. This difference was not statistically significant (p = 0.166). In the group with PD-L1 expression below 80%, there were 19 males (76.0%) and 6 females (24.0%). In the group with PD-L1 expression of 80% or higher, there were 12 males (46.2%) and 14 females (53.8%). The difference in gender distribution between the two groups was statistically significant (p = 0.029). Most patients with PD-L1 values below 80% had a BMI of 25.0-29.9, while the majority of patients with PD-L1 values of 80% or higher had a BMI of 18.5-24.9. There was no statistically significant difference in BMI between the two groups (p = 0.394). In both groups, the majority of patients were smokers, with no statistically significant difference between groups based on smoking status (p = 0.099). Squamous and non-squamous tumor types were equally represented in both groups, and the difference in tumor type based on PD-L1 values was not statistically significant (p = 0.903). Slightly more patients with PD-L1 values below 80% were in stage IVA compared to those with PD-L1 values of 80% or higher (84.0% *vs*. 57.7%), and this difference was statistically significant (p = 0.039). Partial response (PR) was observed in 12 patients (48.0%) with PD-L1 values below 80%, while the same response was observed in 7 patients (26.9%) with PD-L1 values of 80% or higher. The difference in best response between the two groups was not statistically significant (p = 0.262). Disease progression occurred in 12 patients (48.0%) with PD-L1 values below 80%, and in 15 patients (57.7%) with PD-L1 values of 80% or higher. This difference was not statistically significant (p = 0.488). In the group with PD-L1 values below 80%, there were 13 deaths (52.0%), while in the group with PD-L1 values of 80% or higher, there were 17 deaths (65.4%). Again, the difference was not statistically significant (*p* = 0.332). Multivariate analysis of evaluable variable is given in Table 6.

**FIGURE 1.**
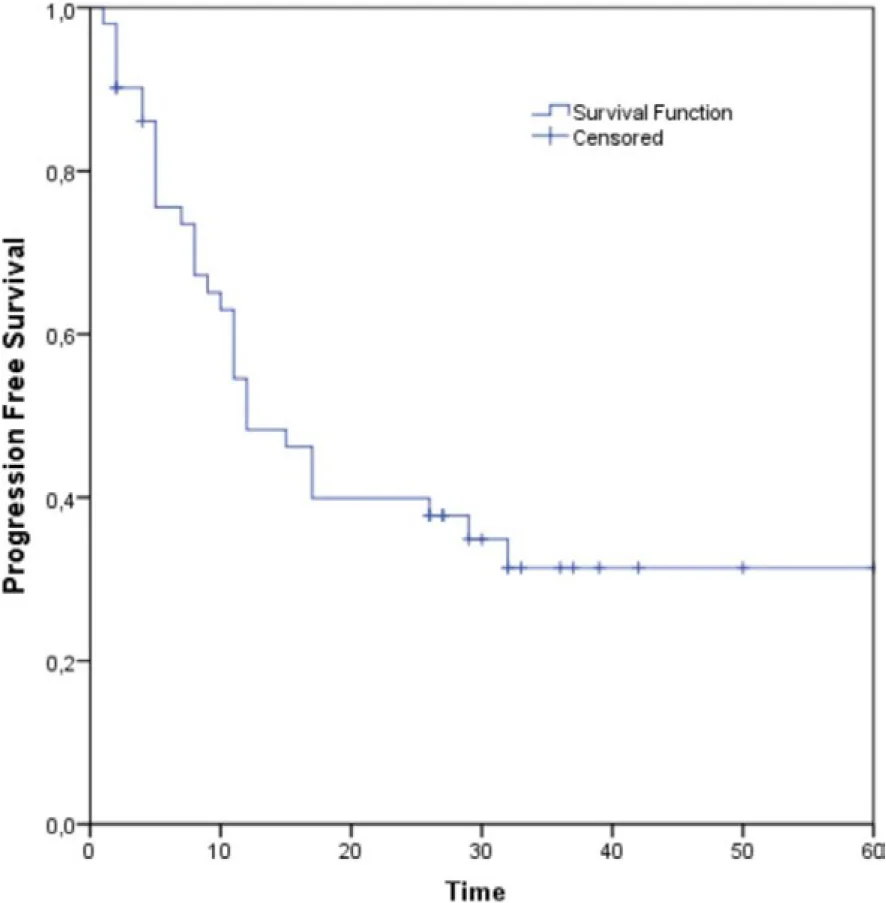
Progression free survival (PFS) for all patients evaluated in the real-world data (RWD) analysis.

**FIGURE 2.**
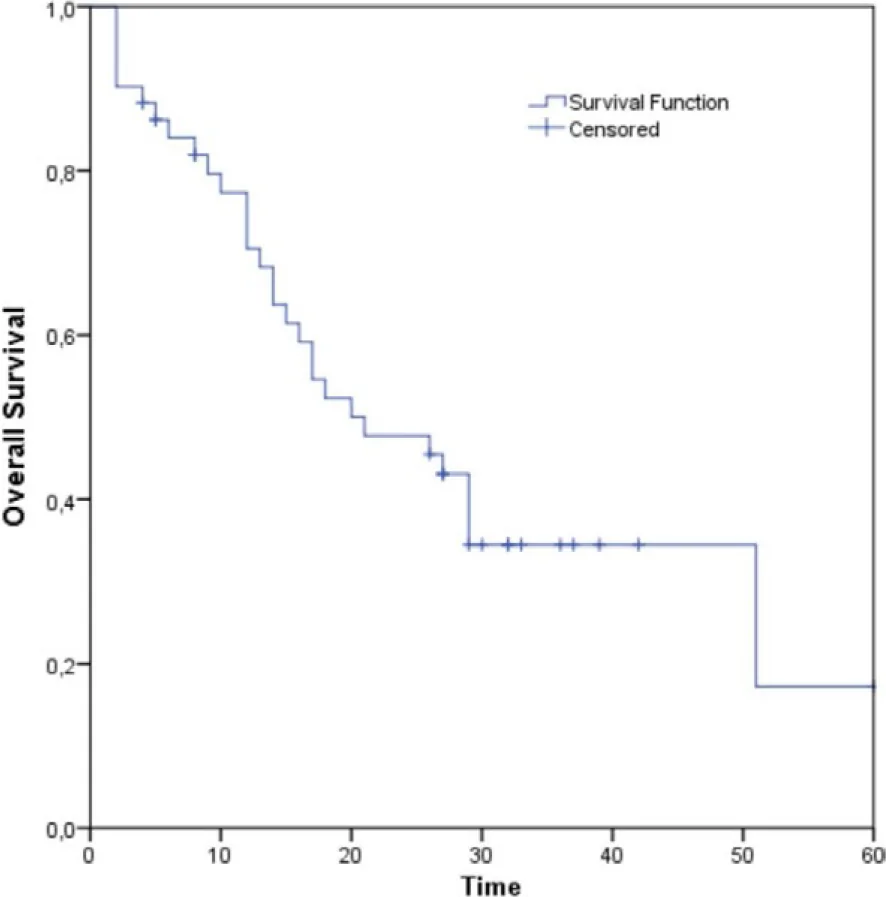
Overall survival (OS) for all patients evaluated in the real-world data (RWD) analysis.

**TABLE 6.** Multivariate analysis of patient characteristics in relation to PD-L1 expression

Characteristic	PD-L1 (50–79%)	PD-L1 (80–100%)	*p* value
**Age (± SD)**	65.4 (7.9)	68.2 (5.8)	*0.166*
**Gender, N (%)**			
Male	19 (76.0)	12 (46.2)	***0.029***
Female	6 (24.0)	14 (53.8)	
**BMI**			
< 18.5	1 (4.0)	2 (7.7)	0.394
18.5–24.9	9 (36.0)	13 (50.0)	
25.0–29.9	12 (48.0)	11 (42.3)	
30.00–34.9	1 (4.0)	0 (0.0)	
35.00–39.9	0 (0.0)	0 (0.0)	
> 40	2 (8.0)	0 (0.0)	
**Smoking status, N (%)**			
Nonsmokers	0 (0.0)	3 (11.5)	0.099
Smokers	22 (88.0)	17 (65.4)	
Former smokers	3 (12.0)	6 (23.1)	
**Type of NSCLC, N (%)**			
Squamous	11 (44.0)	11 (42.3)	0.903
Non-squamous	14 (56.0)	15 (57.7)	
**Stage, N (%)**			
IVa	21 (84.0)	15 (57.7)	**0.039**
IVb	4 (16.0)	11 (42.3)	
**Best response, N (%)**			
PR	12 (48.0)	7 (26.9)	0.262
SD	10 (40.0)	16 (61.5)	
PD	3 (12.0)	3 (11.5)	
**Progression, N (%)**			
No	13 (52.0)	11 (42.3)	0.488
Yes	12 (48.0)	15 (57.7)	
**Outcome, N (%)**			
Alive	12 (48.0)	9 (34.6)	0.332
Deceased	13 (52.0)	17 (65.4)	

1 BMI = Body Mass Index; NSCLC = Non-Small Cell Lung Cancer; PD-L1 = Programmed Death-Ligand 1; PD = Progressive Disease; PR = Partial Response; SD = Stable Disease; ± SD = ± Standard Deviation

**FIGURE 3.**
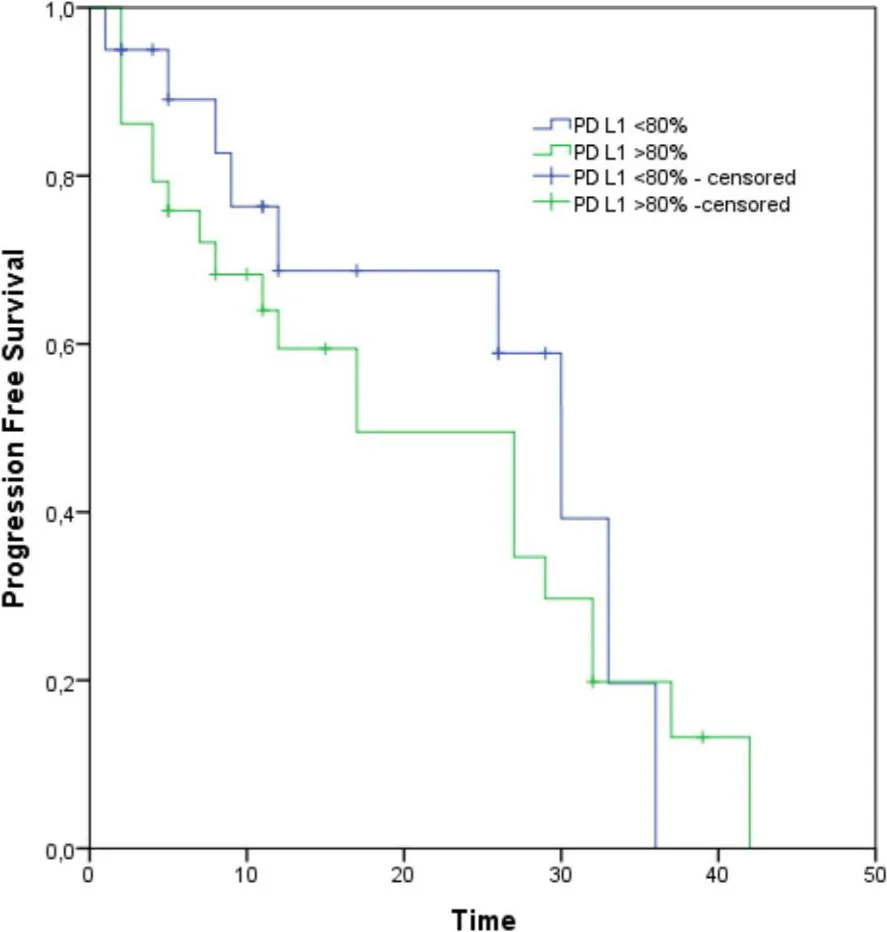
Progression Free Survival (PFS) between PD-L1 ≥ 80% group and PD-L1 < 80%; p = 0.548.

**FIGURE 4.**
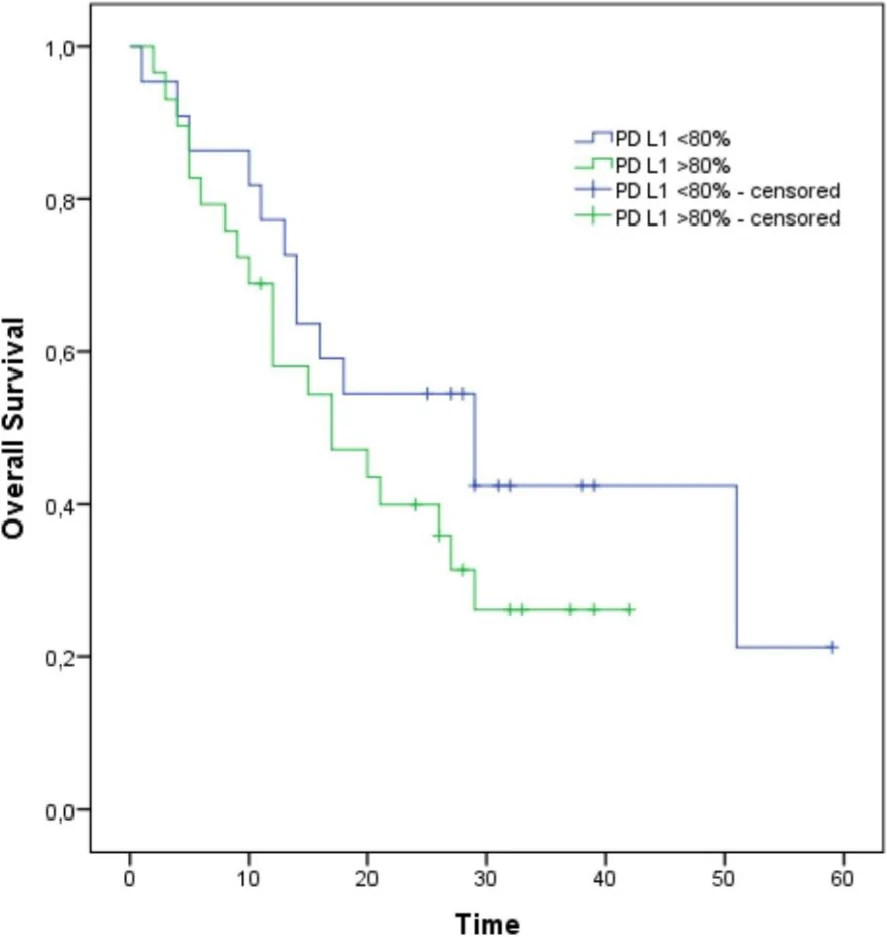
Overall Survival (OS) between PD-L1 ≥ 80% group and PD-L1 < 80%; p = 0.153

The average time to disease progression for patients with PD-L1 expression below 80% was 24.3 months, while for patients with PD-L1 expression of 80% or higher, it was 20.6 months. There is no statistically significant difference in progression free survival between two groups (p = 0.548) (Figure 3). The average overall survival for patients with PD-L1 expression of 50%–80% was 34.0 months, whereas for those with PD-L1 expression of 80%–100%, it was 22.1 months. There is no statistically significant difference in overall survival between these two groups (p = 0.153) (Figure 4). Median follow-up time was 32 months, 95%CI (29.5–34.5).

## Discussion

This trial, which included 51 patients with NSCLC and PD-L1 expression ≥ 50% treated with pembrolizumab in the first-line setting, the median progression-free survival (mPFS) was 12.0 months, and the median overall survival (mOS) was 21.0 months. Among the full cohort in our study, the ORR was 37.2%. Based on a five-year follow-up result of KEYNOTE-024 study, mPFS for pembrolizumab-treated patients was 7.7 months, and mOS was 26.3 months. The objective response rate (ORR) was 46.1%.^15,16^ We observed slightly higher median PFS, while median OS and ORR were somewhat lower compared to KEYNOTE-024. These discrepancies can be attributed to the design of our research, the small sample size, and the lack of precise data regarding subsequent therapies for patients who progressed after first-line pembrolizumab treatment. Additionally, in our study, as per institutional clinical practice, radiological monitoring was conducted every 12 to 16 weeks, a longer interval compared to the usual monitoring schedule in clinical trials. This might explain the slightly longer mPFS observed in our study compared to the mPFS reported in the KN-024 study. In Serbia, pembrolizumab is reimbursed for use in patients with metastatic NSCLC and PD-L1 expression ≥ 50%, and there is no cap on the number of immunotherapy cycles. However, in the clinical study design of Keynote-024, pembrolizumab was administered for a maximum of 35 cycles or earlier if disease progression, significant adverse events, or unacceptable toxicity occurred. A French research group published results of their retrospective study on first-line pembrolizumab treatment in RWD setting, for patients with metastatic NSCLC and PD-L1 expression > 50%.^17^ Their study, like ours, was retrospective RWD study and lacked a control group but included more patients – 118 in total. The mPFS was slightly lower, 10.1 months, but the ORR (58.2%) was significantly higher compared to our study and the ORR observed in the KN-024 trial. Over 50% of patients in the French study showed partial responses (PR), and 2.7% had complete responses (CR). In contrast, none of our patients achieved a CR with pembrolizumab therapy, and only 37% of patients had a PR. In a multicenter retrospective study in Japan (HOPE-001) the main focus was on the efficacy and safety of pembrolizumab monotherapy in real-world practice. This study involved 213 patients predominantly consisted of older male patients (82.6% male, with a mean age of 71 years). The ORR was 51.2% with median progression-free survival of 8.3 months, and the mOS of 17.8 months.^18^ Our study population, even thou smaller in number, was slightly younger (mean age of 66 years) and more balanced by gender (60% male, 40% female). Regarding efficacy, the objective response rate in our study was lower than in the Japanese study; however, both mPFS (12.0 months) and mOS (21.0 months) were longer. The mPFS and mOS in Japanese study were slightly shorter also in comparison with the results from KN-024. These differences might be explained by the older patient population included in Japanise study compared to KN-024 and our research. A large European study involving 1026 patients reported an ORR of 44.5%, a mPFS of 7.9 months, and a mOS of 17.2 months.^19^ This study had an ORR comparable to KN-024 but superior to our study’s ORR. Notably, the European study’smPFS and mOS were shorter than both KN-024 and our study. This difference may stem from the inclusion of patients with ECOG PS 2 (17%), ECOG PS 3 (0.4%), and patients with untreated brain metastases, which are common in everyday real-world clinical practice, but usually excluded from clinical trials. In our study, the exact metastatic disease locations were not precisely documented, representing a limitation. However, no patients with ECOG PS > 1 were included in our study, as the Republic Health Insurance Fund in Serbia only permits pembrolizumab treatment for patients with ECOG PS 0 and 1 in standard clinical practice. Despite these limitations, the efficacy of pembrolizumab in our study aligned well with findings from other real-world studies.^20,21^

Given that PD-L1 is a continuous biomarker correlating with the efficacy of immunotherapy in NSCLC patients, our study sought to determine whether patients with higher PD-L1 expression experienced better PFS and OS outcomes with first-line pembrolizumab therapy. With a median PD-L1 expression of 80% in our cohort, patients were divided into two groups: one with PD-L1 expression of 50%–80% and the other with PD-L1 expression of 80%–100%.

In a study by Aguilar *et al*. clinical outcomes were examined in NSCLC patients with very high PD-L1 expression.^21^ The study conducted by Aguilar and colleagues included 187 patients with NSCLC and PD-L1 expression levels above 50%, which was three times the number of patients in our study. The ORR for the overall patient population in Aguilar’s study was higher than in ours, reaching 44.4%. Median progression-free survival was only 6.5 months, while median overall survival was not reached. The median follow-up time of 12.6 months could explain the lower mPFS and the observed mOS results. In Aguilar’s study, patients were divided into two groups based on PD-L1 expression to evaluate treatment outcomes in individuals with the highest PD-L1 levels. One group consisted of patients with PD-L1 expression levels of 50%–89% (N = 107), while the other included patients with levels of 90%–100% (N = 80). This division was based on the median PD-L1 expression of responders, which was 90%, contrasting with the median PD-L1 expression of 80% in our study. Both groups were well-balanced in terms of age, gender, tumor histology, smoking status, ECOG PS, and KRAS mutation status, with no statistically significant differences in clinical-pathological characteristics between them. In our research, we observed statistically significant differences in gender distribution regarding PD-L1 expression (p = 0.029), and patients with PD-L1 levels below 80% were more frequently in stage IVA compared to those with PD-L1 ≥ 80% (84.0% *vs*. 57.7%, p = 0.039). However, there were no significant differences in age, smoking status, BMI, or tumor type between the two groups in our study.

In Aguilar’s study, patients with PD-L1 expression levels of 90%–100% had an ORR of 60%, while patients with PD-L1 expression levels of 50%–89% had an ORR of 32.7%. This difference was statistically significant (p < 0.001). Current study in our group showed no significant differences in response to pembrolizumab between the two groups, although numerically, patients with PD-L1 expression levels of 80%–100% tended to have stable disease (SD) as the best response, while patients with PD-L1 levels of 50%–80% were more likely to show partial response (PR). This result might be attributed to the smaller patient sample sizes and the different PD-L1 cutoff values used between the two studies. Aguilar’s study found that mPFS was significantly longer in the PD-L1 90%–100% group compared to the PD-L1 50%–89% group (14.5 months *vs*. 4.1 months, p < 0.01). Similarly, mOS was significantly longer in the PD-L1 90%–100% group compared to the PD-L1 50%–89% group (not reached *vs*. 15.9 months, p = 0.002). Comparable results were also seen in Cortellini`s study. The median PFS and the median OS were also significantly shorter among patients with PD-L1 expression of < 90% as compared to those with a PD-L1 expression of ≥ 90%.^19^ Several other studies also showed that patients with very high PD-L1 expression (PD-L1 > 90%) had a clear advantage in terms of mPFS and MOS in comparison to the patients with high PD-L1 expression (PD-L1 50%–90%).^22,23^ In our study, the situation was reversed: both mPFS and mOS were longer in the PD-L1 50%–80% group, although these differences were not statistically significant. This could be explained by several factors, including the small sample size and different PD-L1 thresholds for group selection.

Even though current trial did not show significant difference in survival outcomes for patients whose tumors present expression of PD-L1 over 80% it does not necessarily mean that this prognostic factor should be neglected. The major limitations of this trial are retrospective design and smaller sample. However, that pitfall might be good starting point for piling data from smaller RWD trials, enabling direct comparison between patient populations. On the other hand, the inevitable expansion of our cohort, as well the other cohorts currently recruiting patients in prospective parts of their trials might reveal contrasting data among larger trial population. Anyhow, all RWD data would be necessary for generating real-world evidence which might impact current treatment guidelines.
